# Case report: A retrospective serological analysis indicating human exposure to tick-borne relapsing fever spirochetes in Sonora, Mexico

**DOI:** 10.1371/journal.pntd.0007215

**Published:** 2019-04-11

**Authors:** Edwin Vázquez-Guerrero, Norma P. Adan-Bante, Mónica C. Mercado-Uribe, César Hernández-Rodríguez, Lourdes Villa-Tanaca, Job E. Lopez, J. Antonio Ibarra

**Affiliations:** 1 Laboratorio de Genética Microbiana, Departamento de Microbiología, Escuela Nacional de Ciencias Biológicas, Instituto Politécnico Nacional, Ciudad de Mexico, Mexico; 2 Laboratorio de Investigación en Zoonosis y Enfermedades Tropicales, Unidad Regional Sur, Universidad de Sonora, Navojoa, Sonora, Mexico; 3 Departamento de Infectología Pediátrica del Hospital Civil de Guadalajara “Fray Antonio Alcalde,” Guadalajara, Jalisco, Mexico; 4 Laboratorio de Biología Celular y Molecular de Procariotes y Levaduras, Departamento de Microbiología, Escuela Nacional de Ciencias Biológicas, Instituto Politécnico Nacional, Ciudad de Mexico, Mexico; 5 Departments of Pediatrics and Molecular Virology and Microbiology, National School of Tropical Medicine, Baylor College of Medicine, Houston, Texas, United States of America; Yale University, UNITED STATES

## Description of case

**Fig 1:**
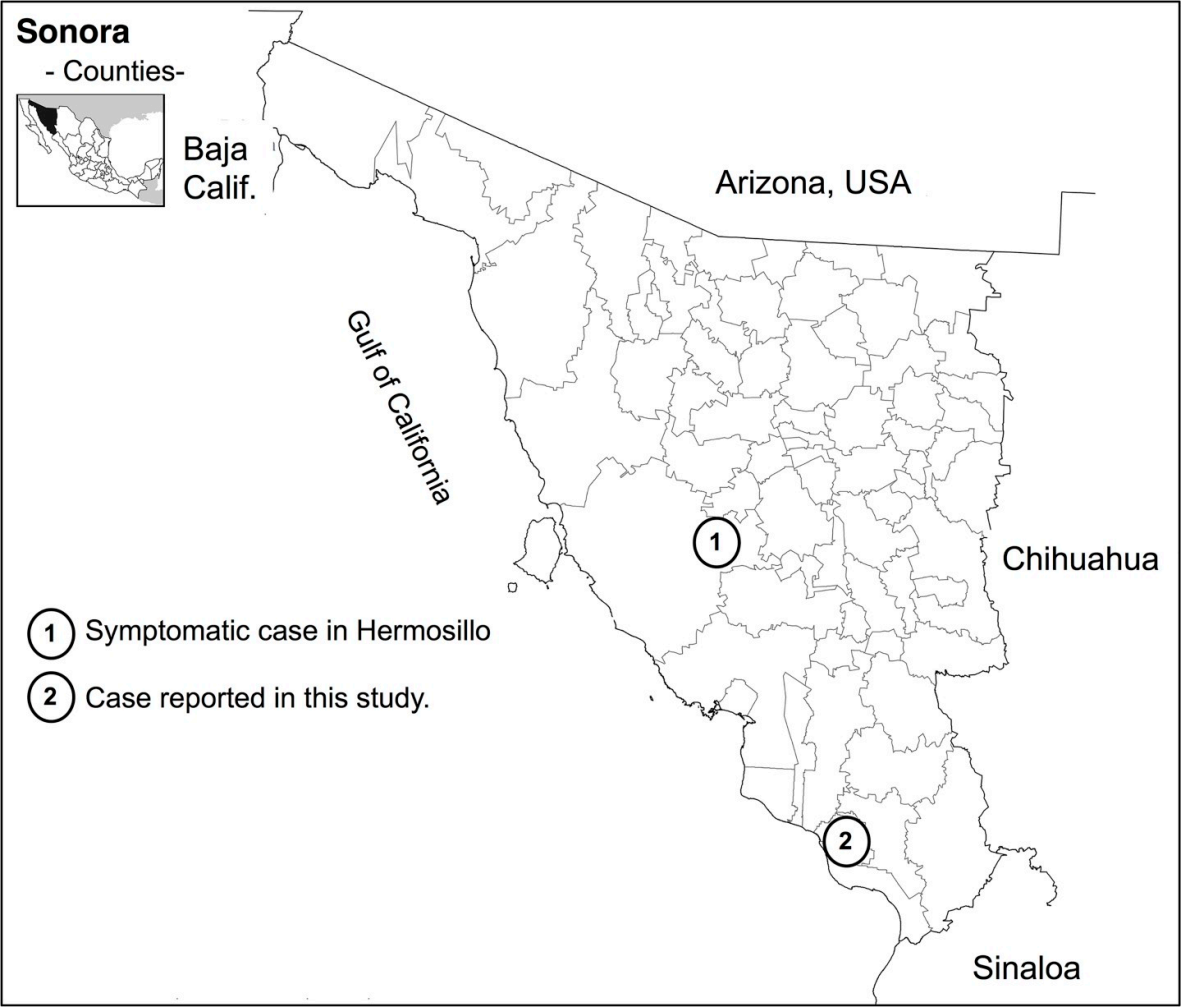
Localization of relapsing fever cases in Sonora, Mexico. Map shows the Mexican state of Sonora, and the inset map in the upper left corner shows the state’s geographical location in the country. Numbers indicate the approximate location of the two described borreliosis cases in Sonora. The map was modified from http://www.beta.inegi.org.mx/app/mapas/?ag=26 and used under the free use terms by INEGI, Mexico. INEGI, Instituto Nacional de Estadística Geografía e Informática.

On January 27, 2012, a 45-year-old woman was admitted for hospitalization. She was from the Bacame Nuevo community, Etchojoa, Sonora, Mexico (Fig 1), a community with a desert climate, dedicated to agriculture and livestock. The patient had a history of outdoor activities and contact with cattle, rodents, dogs, and ticks. She presented with fever, arthralgias, diaphoresis, asthenia, adynamia, fatigue, headache, eye pain, tachycardia, abdominal pain, dyspnea, generalized diffuse rash, insomnia, nocturnal diaphoresis, daytime drowsiness, and character changes that included irritability. During January, she exhibited approximately five febrile episodes of greater than 39°C and was symptomatically treated. She was diagnosed with a fever of unknown origin because she was serologically negative for other common diseases including leptospirosis, syphilis, and Lyme disease. The patient received treatment with beta-lactam antibiotics (amoxicillin for 15 days) without improvement in the symptoms. At her last evaluation, a blood sample was obtained, and spirochetes were observed by dark-field microscopy. She remained serologically negative to leptospirosis, syphilis, and Lyme-disease–causing spirochetes using commercially available diagnostic tests (Leptospira immunoglobulin G [IgG] immunoglobulin M [IgM] ELISA test [Diagnostic Automation, Woodland Hills, CA, USA], Bioelisa Syphilis 3.0 test [Biokit, Lliçà d'Amunt, Spain], and Lyme Disease IgG/IgM ELISA kit [Diagnostic Automation]). The patient was not hospitalized, but she was treated with doxycycline for 14 days and recovered. A retrospective evaluation of this patient’s history and her clinical summary resulted with the suspicion of a tick-borne relapsing fever (TBRF).

*Borrelia* sp. was suspected as the causative agent because of the proximity to the U.S. border and that cases of borreliosis have been identified in regions neighboring the Mexican state of Sonora, specifically Arizona and Texas [1, 2, 3, 4]. Also, during her examination, the patient reported that she found an insect bite on her leg. She had no history of traveling outside of the state of Sonora before the symptoms presented. The tick vectors of *Borrelia turicatae* and *B*. *mazzottii* have been collected along the Texas–Mexico border and into northern Mexico [5, 6]; however, spirochete isolates have not been obtained in culture from Mexico. To determine the patient’s exposure to TBRF spirochetes, a serum sample was evaluated for reactivity against *B*. *turicatae* 91E135 (a Texas isolate that originated from ticks) [7] and to the diagnostic antigen recombinant glycerophosphodiester phosphodiesterase (rGlpQ). This protein is highly conserved among species of relapsing fever spirochetes and is absent in *B*. *burgdorferi*. rGlpQ is an ideal antigen in areas where it remains unclear whether relapsing fever spirochetes are circulating in nature and can be used to discriminate between infections caused by Lyme disease and TBRF spirochetes [8,9]. After the initial screening, the serum sample was further evaluated against the *B*. *turicatae* homologue of the *Borrelia* immunogenic protein A (BipA). While BipA can also discriminate between infections caused by Lyme disease and TBRF spirochetes, evidence indicates that the protein may be a species-specific antigen [10, 11]. Our retrospective serological analysis suggests that the patient was exposed to TBRF spirochetes and that the species may have been *B*. *turicatae*.

## Approach

### Ethics statement

A clinical protocol was approved by the Research Ethics Committee of the Antiguo Hospital Civil de Guadalajara Fray Antonio Alcalde (registry no. 140/17). Also, the patient’s verbal and written informed consent was obtained to test the serum sample. The patient also gave signed consent to have the details published.

### Production of rGlp Q and rBipA

**Fig 2:**
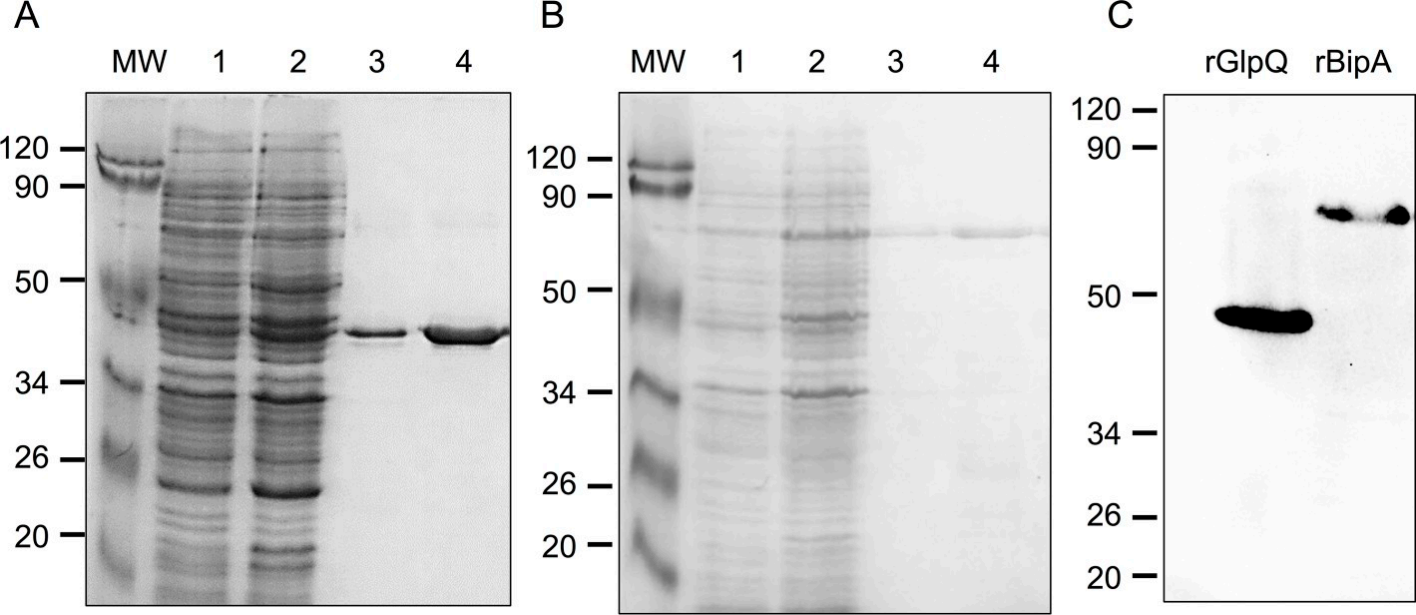
rGlpQ and rBipA expression and purification. *B*. *turicatae glpQ* was expressed as a recombinant fusion protein (A). Lanes 1 and 2 are *E*. *coli* BL21 pLysS pET-19b-*glpQ* before and after induction with IPTG, respectively; lanes 3 and 4 are two purified fractions of rGlpQ. *B*. *turicatae bipA* was also expressed as a recombinant fusion protein (B). Lanes 1 and 2 are *E*. *coli* BL21 pLysS pET-19b-*bipA* before and after induction with IPTG, respectively; lanes 3 and 4 are two fractions of purified rBipA. Immunoblotting was performed using an anti-histidine probe conjugated to HRP for the histidine epitope on rGlpQ and rBipA (C). Molecular masses are shown on the left of each immunoblot in kilodaltons. MW, molecular weight; rBipA, recombinant *Borrelia* immunogenic protein A; rGlpQ, recombinant glycerophosphodiester phosphodiesterase.

Currently, there is not a commercially available serological test for TBRF spirochetes, and GlpQ was initially used as a diagnostic antigen for infection. *B*. *turicatae* (91E135 isolate) [7] GlpQ was produced as a 10-histidine-residue–linked recombinant protein. To obtain recombinant GlpQ (rGlpQ), pET-19b-*glpQ* expression vector was electroporated into *Escherichia coli* BL21 pLysS. The gene was induced with 1 mM IPTG, and overexpression was verified by SDS-PAGE using a 12% gel (Fig 2A). rGlpQ was purified by immobilized metal affinity chromatography using an Ni-NTA agarose resin column (Jena Bioscience, Löbstedter, Germany). Similarly, recombinant BipA (rBipA) was also overexpressed and purified (Fig 2B). Purification of the proteins was confirmed by western blot by using an anti-histidine probe conjugated to HRP (Pierce Biotechnology, Thermo Fisher Scientific, Rockford, IL, USA) (Fig 2C). The molecular masses of native GlpQ and rGlpQ are 39 and 48 kDa, respectively, while those of native BipA and rBipA are 60 and 70 kDa, respectively.

### Serological evaluation of the patient’s serum sample

**Fig 3:**
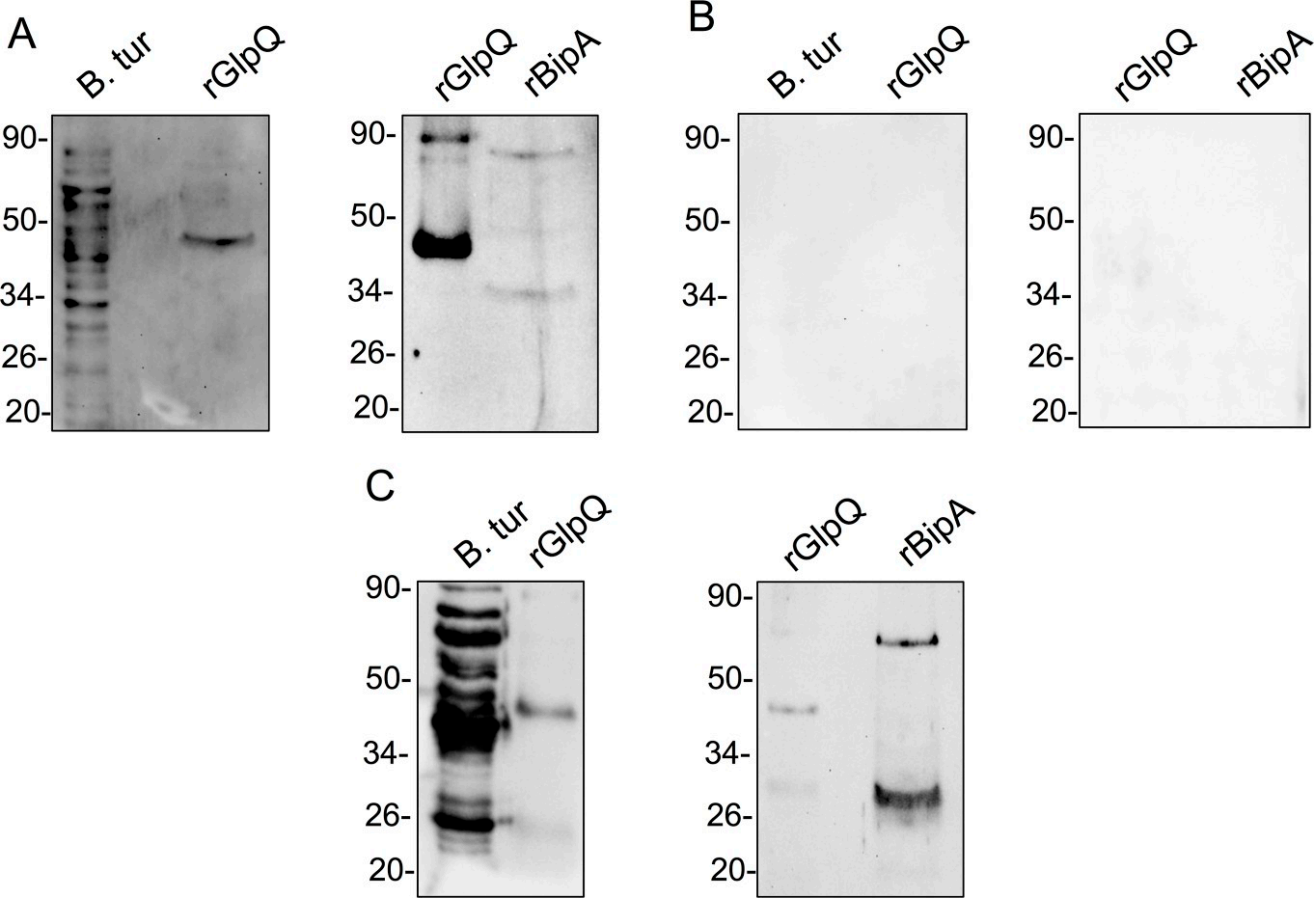
Detection of *B*. *turicatae* and rGlpQ by western blot. The patient’s serum sample was used for immunoblotting using *B*. *turicatae* (B. tur) protein lysate, purified rGlpQ, and rBipA (A). A patient’s serum sample without a clinical history of relapsing fever or contact with the vector was used as a negative control against the same antigens (B). Also shown is reactivity of a positive control serum sample against *B*. *turicatae* protein lysate, rGlpQ, and rBipA (C). Molecular masses are shown on the left of each immunoblot in kilodaltons. rBipA, recombinant *Borrelia* immunogenic protein A; rGlpQ, recombinant glycerophosphodiester phosphodiesterase.

Protein lysates from 1 × 10^7^ spirochetes (the 91E135 isolate of *B*. *turicatae*) and approximately 1 μg of rGlpQ and rBipA were separated by electrophoresis using 12% SDS-PAGE gels and the Mini-PROTEAN 3 System (BioRad, Hercules, CA, USA). Proteins were transferred to polyvinylidene difluoride (PVDF) membranes as previously described using the Amersham WB transfer system (GE Healthcare Life Sciences, Marlborough, MA, USA) [11]. Immunoblots were tested with the patient's serum sample at a dilution of 1:200, using previously reported methods [11], and HRP-rec-Protein G (Invitrogen, Carlsbad, CA, USA) diluted 1:4,000 was used as the secondary molecule. The membrane was developed with a Novex chemiluminescence kit (Invitrogen), and reactivity visualized on a Chemidoc Touch device (Bio Rad). Reactivity to numerous proteins in the *B*. *turicatae* lysate and rGlpQ were observed by immunoblotting (Fig 3A, first panel), indicating that this patient had been exposed to relapsing-fever–causing spirochetes. The serum sample was then evaluated for reactivity to rGlpQ and rBipA (Fig 3A, second panel), which suggested exposure to *B*. *turicatae*. A serum sample from a volunteer without a suspected history of exposure to TBRF spirochetes was used as a negative control (Fig 3B). In a subsequent experiment, a serum sample from a canine infected with *B*. *turicatae* was used as a positive control [11] because positive human controls were unavailable (Fig 3C). In both the patient’s and the canine’s immunoblots, a lower molecular weight band appeared with rBipA, which was likely degraded recombinant protein. Regardless, since neither protein was observed with the negative control, the results suggested that the patient was infected with *B*. *turicatae*.

## Case discussion

Since initial reports in the 1950s of TBRF spirochete transmission from *O*. *turicata* and *O*. *talaje* recovered from Mexico [12], the pathogens have received little attention in the country. Moreover, it remains unclear whether hard tick species of TBRF spirochete are emerging in Mexico, for example, *B*. *miyamotoi*. In 2012, a case report of suspected borreliosis was described in Sonora, Mexico [1]. In that study, a 12-year-old patient was admitted several times to the hospital because of a recurrent fever. Her serological test was negative for Lyme-disease–causing *Borrelia*, but spirochetes were observed in a blood smear, and she was diagnosed with a non-Lyme borreliosis. The patient received antibiotic treatment that included erythromycin, penicillin G, and doxycycline, and no further confirmation was made. In our current study, the patient initially received treatment with amoxicillin, which failed to resolve the infection. While TBRF spirochetes are susceptible to the beta-lactam group of antibiotics, evidence exists that the pathogens may be resistant to amoxicillin [13]. These findings indicate that in regions where there is poor understanding within the medical community of TBRF, improper diagnosis can delay adequate treatment.

In recent years, studies in Mexico reported a Lyme-like disease, but little attention has focused on definitive molecular typing of the *Borrelia* species [14, 15]. In our current case report, the patient’s serum sample was negative for Lyme-disease–causing spirochetes. However, the visualization of spirochetes by dark-field microscopy indicated TBRF spirochetes because the pathogens attain high densities in the blood, as opposed to Lyme-disease–causing *Borrelia* [16]. Importantly, our serological evaluation further supported the identification of an endemic focus for TBRF spirochetes in Sonora, Mexico.

Given the absence of commercially available point-of-care diagnostic tests for TBRF spirochetes, assessing serological responses to rGlpQ and rBipA is an accurate approach to determine exposure to the pathogens. Schwan and colleagues first identified GlpQ as an antigen that can discriminate between infections caused by Lyme disease and relapsing fever spirochetes. The protein is also highly conserved among species of relapsing fever spirochetes [8] and should be used for serological surveillance studies in regions where the circulation of the spirochetes is unclear. Moreover, a previous study indicated that IgM responses to rGlpQ were detectable within 4 days of the spirochete infection [10], suggesting that the protein can be targeted to diagnose early infection. A potential limitation of rGlpQ as a diagnostic antigen is that there are homologues in other pathogens, for example, *Haemophilus influenzae* [10]. The potential of serological cross-reactivity caused us to further evaluate antibody responses to rBipA, and our findings suggest that this patient was infected with *B*. *turicatae*.

To further define the circulation of TBRF spirochetes in Mexico, we recommend increased surveillance efforts to collect *Ornithodoros* ticks and identify the vertebrate hosts involved in maintaining the pathogens in nature. Given the patient’s limited travel history, it is likely that she was exposed to TBRF spirochetes while performing daily work and leisure activities around her home. Field studies should be focused at the suspected exposure site to collect serum samples from small and medium-sized mammals, including rodents and domestic and wild canids. These mammals likely have a role in the ecology of TBRF spirochetes [17]. Furthermore, while most *Ornithodoros* sp. whose biology has been studied are active nocturnally [5, 18], in the USA, we have used carbon dioxide to bait *O*. *turicata* throughout the day [19]. Our studies suggest that vector feeding is likely driven by the detection of host availability. Currently, we are attempting to collect *Ornithodoros* ticks to determine their activity during the day, and future work will focus on peridomestic settings, as we previously reported [6, 19]. Collected specimens will then be used for xenodiagnosis in laboratory animals. As surveillance studies are conducted from suspected exposure sites, a refined understanding will be attained regarding the maintenance and emergence of TBRF spirochetes in Mexico.

### Key learning points

- A case of borreliosis in northern Mexico was confirmed.
- The rGlpQ and rBipA suggested the diagnosis of *B*. *turicatae* as the causative agent.
- Increased surveillance efforts with at-risk populations will aid in the diagnosis and treatment of patients and will also refine the understanding of the prevalence and emergence of the pathogens in Mexico.
